# Case Report: Incidental finding of a perforating wooden foreign body in the jejunum during surgical treatment of colic in a horse: use of intraoperative infrared thermography

**DOI:** 10.3389/fvets.2026.1911195

**Published:** 2026-10-28

**Authors:** Celeste Nanci, Giovanni Biglietti, Ismael Hernández Avalos, Barbara Garbagnati, Pier Luigi Liguori, Maria Rizzo, Claudia Giannetto, Claudia Interlandi

**Affiliations:** 1 Department of Veterinary Sciences, University of Messina, Polo Universitario Annunziata, Messina, Italy; 2 Clinica Veterinaria “Piola”, Milano, Italy; 3 Clinical Pharmacology and Veterinary Anesthesia, Biological Sciences Department, Facultad de Estudios Superiores Cuautitlán, Universidad Nacional Autónoma de Mexico (UNAM), Cuautitlán, Mexico

**Keywords:** colic, enterectomy, foreign body, horses, infrared thermography, jejunal perforation, tissue perfusion

## Abstract

**Background:**

Equine gastrointestinal tract perforation by a foreign body is a rare cause of colic. Assessment of intestinal viability during exploratory laparotomy is crucial and is usually performed using subjective parameters (color, motility, pulse). Infrared thermography (IRT) offers an objective, noninvasive method for assessing tissue surface temperature in real time, as it correlates with local blood perfusion.

**Case presentation:**

In this case, a 13-year-old Italian Saddle mare was admitted with a 24-h history of acute, progressive abdominal pain unresponsive to medical therapy. Clinical examination revealed severe discomfort, systemic compromise (HR: 53 bpm, CRT > 3 s), marked leukocytosis (16,600 cells/mm^3^), hyperproteinemia (8 g/dL), and severe hyperlactatemia (15 mmol/L). Transabdominal ultrasound confirmed small intestinal distention. Emergency exploratory laparotomy revealed a 10 cm solid, irregular mass in the jejunum, causing obstruction and localized perforation. Intraoperative infrared thermography (FLIR ThermaCam E8) was used at three time points (T0: exteriorization, T1: margin selection, T2: pre-repositioning) to monitor surface temperature distributions. At T0, a distinct thermal gradient (Delta *T* = 1.9 °C) was detected between the viable intestinal segments (mean *T* = 35.1 °C) and the affected ischemic area (mean *T* = 33.2 °C). Based on the clinical examination and findings, a segmental enterectomy with end-to-end anastomosis was performed. Post-resection mapping (T2) confirmed equal perfusion of both stumps with a difference between measurements of: Delta *T* = 0.1 °C. Dissection of the resected mass revealed a wooden stick. The mare recovered uneventfully and was discharged 10 days after surgery.

**Conclusion:**

This case demonstrates that foreign body-associated intestinal perforation should be considered among the differential diagnosis of horses presenting with severe small intestinal colic. Furthermore, intraoperative infrared thermography proved to be an adjunctive method for real-time quantification of surface temperatures in the examined stumps, providing an objective assessment of surface temperature patterns to complement visual inspection during tissue evaluation.

## 1 Introduction

Foreign body perforation of the gastrointestinal tract is an uncommon cause of colic in horses. Only a limited number of cases have been reported in the literature (1–3). Clinical presentation is typically characterized by signs of abdominal pain, with or without endotoxemia, depending on the presence and severity of septic peritonitis. When horses fail to respond to conventional medical treatment, including fluid therapy, antimicrobials, and anti-inflammatory or anti- endotoxic therapy, an exploratory laparotomy is indicated (3–5). During surgical intervention, assessment of intestinal viability is critical in determining the need for intestinal resection and anastomosis. Traditionally, surgeons rely on subjective parameters such as intestinal color, motility, mesenteric arterial pulsation, and serosal temperature (6). However, the macroscopic appearance of the intestine may not accurately reflect mucosal integrity or microvascular perfusion (7). As emphasized by Freeman et al. (8), preservation of mucosal viability is the principal determinant of postoperative survival in horses with small intestinal disease. Infrared thermography (IRT) is a non-invasive and reproducible imaging modality capable of assessing tissue surface temperature in real time with minimal interference to the surgical field (9, 10). Because tissue temperature is closely associated with local blood perfusion, IRT may provide additional objective information regarding intestinal viability. Warm intestinal loops generally indicate preserved microvascular perfusion, whereas persistently hypothermic areas may suggest irreversible ischemic injury and necrosis (11). The basis of IRT is detecting infrared radiation (heat) emitted by the surface of a tissue or organism, with a thermal camera producing a false-color image to display surface temperatures. Some pathways of heat exchange in organisms involve cellular metabolism and local blood flow, which help maintain relatively stable body temperature. However, this process can be influenced by physiological or pathological changes that cause surface temperature to rise or fall, with regulation mediated by vasoconstriction and vasodilation via the autonomic nervous system (ANS). IRT technology is a non-invasive method that captures the release of surface heat as images. Increased parasympathetic activity leads to the release of vasodilatory substances such as prostaglandins, bradykinin, and histamine, whereas neurosecretion of catecholamines triggers sympathetic activation, resulting in vasoconstriction. In the first scenario, surface temperatures will be high; in the second, they will decrease. IRT detects heat and blood flow variations to identify underlying inflammation or circulatory issues linked to various conditions. Abdominal IRT offers a valuable intraoperative or diagnostic way to identify gastric and intestinal problems, such as Inflammatory Bowel Disease, where hyperthermic regions indicate active inflammation associate with the release pro-inflammatory mediators such as prostaglandins, prostacyclin, interleukins (IL), leukotrienes, cytokines, histamine, serotonin, bradykinin, and alpha tumor necrosis factor (TNF-α), among others, which induce vasodilatation, increase blood flow and permeability of blood vessels, emigration of leukocytes, migration by chemotaxis to the injury site, and the release of chemical mediators, causing signs such as redness, swelling, pain, loss of function, and local heat (12). However, despite its potential, the clinical translation of intraoperative IRT requires careful interpretation, as thermographic imaging evaluates only surface radiated heat rather than providing a direct measurement of microvascular perfusion. Surface temperature can be significantly influenced by multiple confounding physiological and environmental factors such as local vasomotor responses, reactive hyperemia, evaporative cooling, tissue exposure duration, and variable camera-to-tissue geometry. Furthermore, the current lack of standardized diagnostic thresholds, reference values, and direct histological correlations in equine abdominal surgery underscores the need for a nuanced approach, viewing IRT strictly as a relative indicator of thermal gradients rather than an absolute diagnostic standard. In veterinary research, IRT is used to monitor tissue health during intestinal surgeries, especially to assess blood flow disruptions caused by foreign body obstructions that squeeze the intestinal walls, leading to ischemia, inflammation, and potential tissue death. Clinically, IRT has shown that in intestinal obstruction cases, the small intestine's surface temperature is notably lower typically by 2.4 °C to over 5 °C compared to healthy, well-perfused tissue (9, 13, 14). This report describes an unusual case of jejunal perforation caused by a wooden foreign body in a horse presented with acute colic. In addition, the case highlights the potential utility of intraoperative infrared thermography as an adjunctive tool for evaluating intestinal perfusion and supporting surgical decision-making.

## 2 Case description

### 2.1 Clinical history

A 13-year-old Italian Saddle mare weighing 422 kg was referred to the clinic for evaluation of acute abdominal pain. The anamnesis revealed that the onset of clinical signs had started the previous day and included inappetence and intermittent restlessness. On the morning of admission, the mare had been treated on the farm with N-butylscopolammonium bromide and metamizole sodium administered at a dose of 0.05 ml/kg, delivering approximately 25 mg/kg of metamizole sodium and 0.2 mg/kg of N-butylscopolammonium bromide (Buscopanvet compositum fl 100 ml, Boehringer Ingelheim Animal Health Italia S.p.A.), followed by flunixin meglumine 1.1 mg/kg (Meflosyl 50 mg/ml, Zoetis S.r.l, Italy) administered intravenously. Due to progressive worsening of the clinical condition and persistence of pain despite medical treatment, the mare was referred to the clinic for further evaluation and possible surgical intervention.

### 2.2 Physical exam

At admission, the mare was depressed and showed signs of severe abdominal discomfort. Physical examination revealed a heart rate (HR) of 53 beats/minute, respiratory rate (RR) of 30 breaths/minute, congested mucous membranes, prolonged capillary refill time (CRT > 3 s), and a rectal temperature of 38.8 °C. Abdominal auscultation revealed markedly reduced borborygmi in all four abdominal quadrants. Rectal palpation identified multiple distended loops of small intestine, whereas no abnormalities were detected in the colon or cecum. Hematologic and biochemical analysis at admission demonstrated marked leukocytosis (16,600 cells/mm^3^), packed cell volume of 35%, total plasma protein concentration of 8 g/dL, and severe hyperlactatemia (15 mmol/L). Based on the heart rate, respiratory rate, leukocytosis, and elevated lactate levels, the patient met the clinical criteria for Systemic Inflammatory Response Syndrome (SIRS) and substantial systemic compromise. Transabdominal ultrasonography revealed multiple markedly distended loops of small intestine within the ventral abdomen and inguinal region. Although these alterations reflect a major systemic inflammatory response, the lack of specific inflammatory markers (such as serum amyloid A or fibrinogen) and peritoneal fluid analysis prevents definitive differentiation between infectious/septic involvement and non-septic systemic inflammation. Based on the overall clinical findings, abdominal ultrasonography, and blood tests, an exploratory laparotomy was immediately indicated. The differential diagnosis primarily included a strangulating lesion of the small intestine.

### 2.3 Pain assessment

Pain was assessed using the Horse Grimace Scale (HGS), with a maximum score of 12 based on six Facial Action Units (FAUs). All assessments were independently performed by three observers, and inter-observer reliability showed a significant level of agreement (Kendall's coefficient of concordance, *p* = 0.001). Measurements were recorded at admission, 8 h post-operatively, and 24 h post-operatively.

### 2.4 Infrared thermography (IRT)

Thermal images were captured by a PhD researcher using a digital infrared thermal camera (ThermaCam E8, FLIR Systems, Boston, MA, USA). All images were acquired at a fixed distance of 1 m from the intestinal surface and at an approximately 90° angle to the site of interest in order to minimize environmental variability. Camera settings included a temperature range between 10 and 40 °C; emissivity of 0.98; a reflected air temperature (Trifl) of 20 °C; a distance (Dist) of 1 m; and a field of view (FOV) of 23°. The detector consisted of an uncooled microbolometer with a focal plane array (FPA), featuring a resolution of 320 × 240 pixels, a thermal sensitivity of 0.08 °C at 30 °C, an (IFOV) of 1.3 mrad, a spectral range from 7.5 to 13 μm, and an accuracy of ±2 °C. The average temperature within each ellipse is calculated by the software. Automatic environmental corrections were applied according to reflected ambient temperature, distance, relative humidity, and atmospheric transmissivity. Thermal measurements were performed at three time points: T0 (Baseline): immediately after exteriorization of the affected intestinal segment; T1 (Intraoperative phase): corresponds to assessment of the margins and resection segments of the enterectomy; and T2 (Pre-repositioning phase): final assessment before replacement of the intestinal loops into the abdominal cavity.

### 2.5 Surgical procedure

Before the procedure, Ringer's lactate solution was administered at a rate of 8 mL/kg/h (Ringer's Lactate 5 Liters Salf, Farmax, Italy) for hemodynamic stabilization and to initiate the administration of other medications. Therefore, 25 mL of benzylpenicillin procaine 11,850 IU/kg and dihydrostreptomycin 15 mg/kg (RePen 250 mL, Fatro, Italy) were administered intramuscularly before surgery. The anesthetic protocol consisted of premedication with xylazine 0.5–1.1 mg/kg, IV (Rompun 2%, Bayer, Leverkusen, Germany) in combination with butorphanol tartrate 0.01 mg/kg, IV (Dolorex 10 mg, Codifa, MSD Animal Health S.r.l., Italy). Anesthesia was induced with ketamine 2.0 mg/kg, IV (Ketavet 100, MSD Animal Health S.r.l., Italy ) and diazepam 0.05 mg/kg, IV (Diazedor 5 mg/ml, TREI S.p.A. a Livisto company, Italy), and maintained with isoflurane (Vetflurane 1,000 mg/g Virbac S.r.l., Milano, Italy) delivered in 100% oxygen. During surgery continuous rate infusion (CRI) of 2% lidocaine (Lidocaina Cloridrato Esteve 2%, Ecuphar Italia S.r.l., Milan, Italy) was administered. The protocol starts with a loading dose of 1.3 mg/kg delivered over 10 min, followed by a maintenance rate of 3 mg/kg/h for the duration of the procedure. During the surgery, dimethyl sulfoxide 0.13 g/kg (DMSO 99.5%, ACEF, Italy) diluted in 500 mL of saline solution (0.9% Sodium Chloride 5 lt Bag V G.S.) was administered as an intravenous infusion to reduce systemic inflammation and provide antioxidant protection. Exploration of the abdominal cavity revealed normally positioned intestinal loops; however, a firm, irregular mass approximately 10 cm in diameter was identified involving the affected jejunal segment and its mesentery. The obstructing mass had an uneven surface. No generalized peritonitis, free fluid, or severe peritoneal contamination was observed in the abdominal cavity. The surrounding tissues exhibited a localized chronic inflammatory reaction with minimal fibrin deposition and adhesions strictly confined to the site of the lesion, thereby completely preventing leakage into the abdominal cavity. The affected jejunal segment containing the mass was exteriorized through the incision and isolated from the abdominal cavity, other normal viscera, the rest of the abdominal contents, and the skin incision (Figure 1). Intestinal viability was assessed by the surgeon subjectively based on serosal color, mesenteric arterial pulsation, intestinal motility, and wall temperature. Intraoperative radiometric images (IRT) were acquired during surgical exploration (Figure 2). Ambient temperature ranged from 20 °C to 24 °C, with relative humidity approximately 60%. The surgeon was asked to pause for a few seconds between acquisitions to minimize the possibility of distortions or artifacts in the thermal recording (Table 1). At baseline (T0), the viable jejunal stump showed a mean temperature (*T*_mean_) of 35.1 °C, whereas the affected stump showed a lower *T*_mean_ of 33.2 °C, with a thermal gradient (Δ*T*) of 1.9 °C between the two segments. During assessment of the enterectomy margins (T1), the viable stump had a *T*_mean_ of 34.6 °C compared with 34.2 °C in the selected stump, resulting in a Δ*T* of 0.4 °C. Based on the intraoperative assessment, including the IRT findings, the selected stump was considered suitable as the resection margin. Before intestinal repositioning (T2), the *T*_mean_ values were 34.8 °C for the viable stump and 34.7 °C for the selected stump, with a further reduction of the thermal gradient to 0.1 °C. The corresponding maximum and minimum temperatures at each time point are reported in Table 1. The mass was removed via segmental resection, performed proximally and distally to the obstruction site, preserving adequately perfused tissue margins. The anastomosis was then performed with assistance that held the ends of the intestine in apposition, using a simple full-thickness continuous suture with Surgicryl 910 (USP 2/0) EP 3 (1/2 circle, 30 mm, Braided Synthetic Absorbable Surgical Suture). Following copious lavage with warm sterile saline solution, the intestinal tract was repositioned into the abdominal cavity. This was followed by further rinsing and the administration of ampicillin, injected directly into the abdominal cavity at a dose of 0.02 g/Kg (10 g/50 mL Vetamplius, Fatro, Italy). During the postoperative period, supportive therapy was administered: benzylpenicillin procaine 11.850 IU/kg and dihydrostreptomycin 14.8 mg/kg l (RePen 250 mL, Fatro, Italy) were administered intramuscularly every 24 h for 5 days; and flunixin meglumine 1.1 mg/kg (Meflosyl 50 mg/ml, Zoetis S.r.l, Italy) was administered intravenously every 12 h for 3 days. Additionally, calcium heparin approximately 89 IU/kg (Calcieparina 12.500 IU/0.5 ml Italfarmaco, S.p.A.; Italy) was initiated immediately post-operatively to prevent thromboembolic complications and mitigate the risk of disseminated intravascular coagulation (DIC), and was administered twice daily for 3 days. Rehydration was maintained through a continuous infusion of Ringer's lactate (Ringer's Lactate 5 Liters Salf, Farmax, Italy). The HGS score was 1.7 at admission, decreasing to 0.7 at 8 h post-surgery, and 0.3 at 24 h. This progressive reduction in pain scores mirrored the clinical recovery following the surgical resolution of the obstruction and the administration of perioperative and postoperative analgesic treatments. The mare was discharged in good health 10 days later. The horse was fed hay in small amounts, starting at 18– 24 h after surgery, and this was gradually increased to full feed over the next 3 days. According to a long-term telephone follow-up with the owner and stable manager, the mare resumed normal feeding and returned to regular riding activities within a few weeks after discharge. No recurrence of colic or other postoperative complications has been reported, and the mare remains in excellent clinical health 13 months following the surgery (Figure 3) The resected mass was subsequently opened, revealing a foreign body of vegetal origin (a small wooden stick) embedded in the intestinal wall (Figure 4).

**Figure 1:**
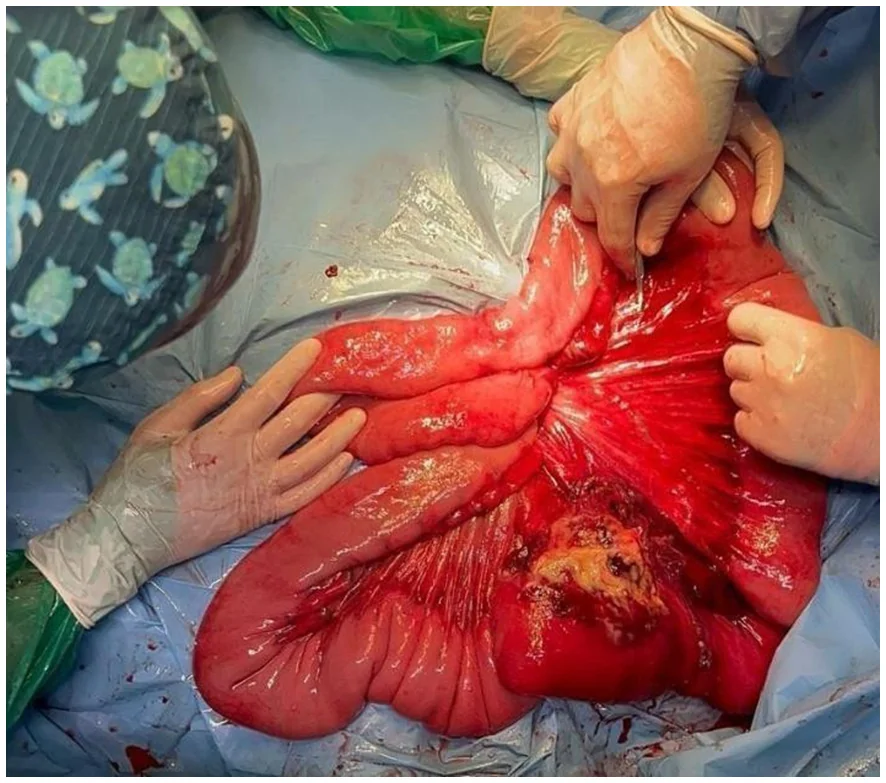
Image of the intestine showing a solid obstructive mass with an irregular surface approximately 10 cm in diameter, involving the jejunum and mesentery.

**Figure 2:**
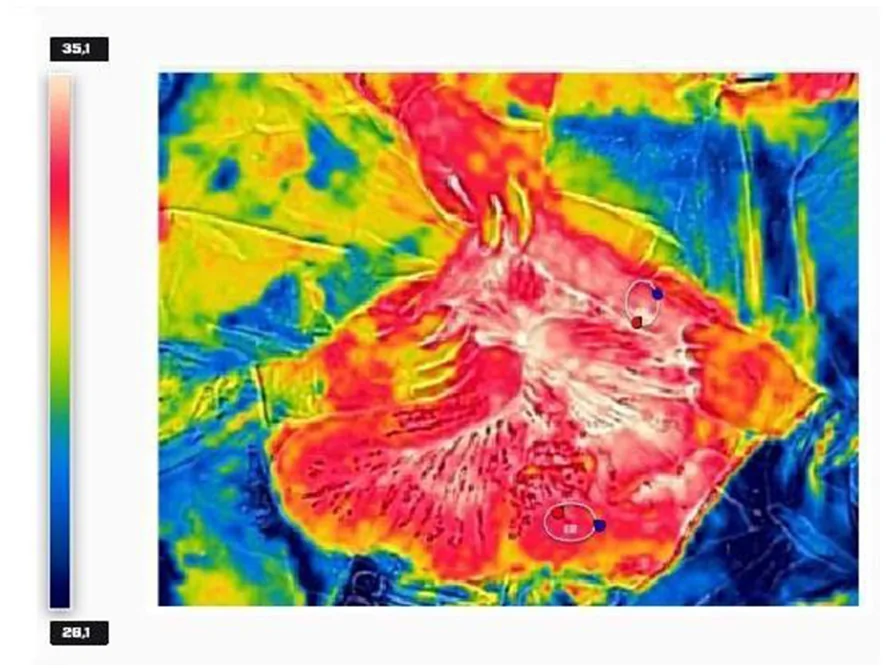
Infrared thermographic image of obstructive mass and evaluation acquired intraoperatively during surgery.

**Table 1:** Intraoperative infrared thermographic assessment of intestinal segments during surgical exploration at three different intraoperative time points: baseline assessment immediately after exteriorization of the affected jejunal segment (T0), assessment of the margins and resection segments of the enterectomy (T1), and before intestinal repositioning into the abdominal cavity (T2).

Time points	Intestinal segment	*T*_max_ (°C)	*T*_min_ (°C)	*T*_mean_ (°C)	Δ*T* (°C)
T0	Viable stump	35.8	34.2	35.1	1.9
	Affected stump	34.4	32.1	33.2	
T1	Viable stump	35.5	34.2	34.6	0.4
	Selected stump	34.9	33.8	34.2	
T2	Viable stump	35.7	33.9	34.8	0.1
	Selected stump	35.1	34	34.7	

Values are reported as maximum temperature (*T*_max_), minimum temperature (*T*_min_), mean temperature (*T*_mean_), and thermal gradient (Δ*T*) between evaluated intestinal segments.

**Figure 3:**
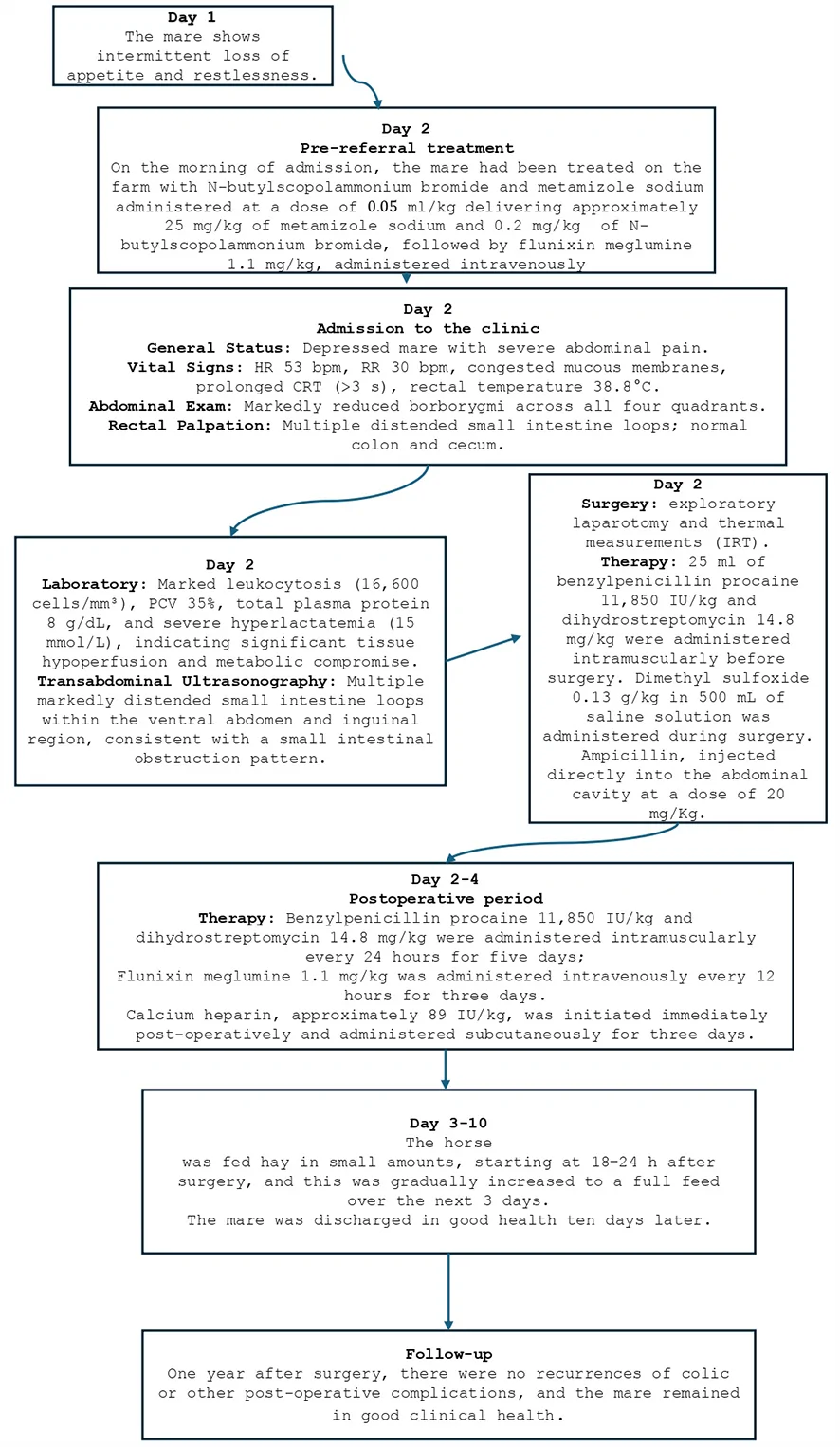
Flow diagram showing the chronological sequence of the clinical management.

**Figure 4:**
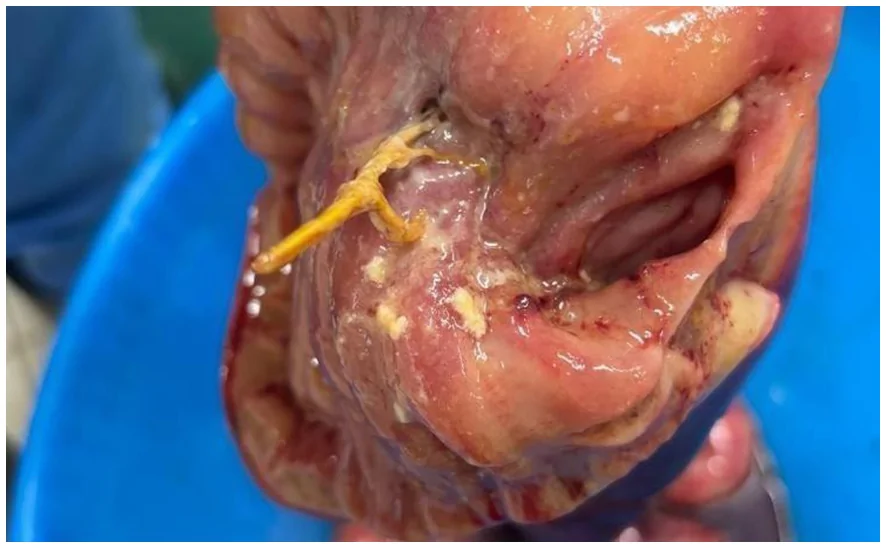
The resected intestinal segment is dissected; a foreign body of vegetal origin (small wooden stick) is identified.

## 3 Discussion

This clinical case highlights the potential application of intraoperative infrared thermography (IRT) as a complementary tool during equine abdominal surgery for a jejunal foreign body perforation, while also underscoring the critical methodological and technical limitations associated with its use. The selection of intestinal segments for anastomosis was performed in accordance with the clinical evaluation methodology proposed by Freeman et al. (8), incorporating conventional intraoperative criteria for intestinal viability. IRT was subsequently used as a complementary imaging technique during the assessment of the selected intestinal segments. The selected stumps showed comparable surface temperatures on the final thermographic assessment. Although these thermal results suggest adequate surface perfusion to support intraoperative decision-making, the lack of histopathological confirmation remains a limitation, preventing definitive conclusions regarding microscopic tissue viability, ischemia, or microvascular status. Indeed, IRT detects infrared radiation emitted from the tissue surface and provides a non-invasive representation of surface temperature. Because tissue temperature is influenced by several factors, including local blood flow, metabolic activity, vasomotor responses, tissue exposure, and environmental conditions, changes in surface temperature may provide indirect information related to local tissue perfusion and viability (10, 15–19). However, IRT does not directly measure blood flow or microvascular perfusion. Therefore, IRT results should be interpreted as a complementary clinical tool rather than a definitive diagnostic standard for tissue pathology (16–18). It is capable of indirectly assessing the integrity of superficial peripheral blood vessels and tissue viability by monitoring radiated heat. This technology enables the early identification of alterations in superficial blood perfusion associated with various pathologies, offering promising support in both clinical practice and experimental settings (19, 20). Infrared thermography (IRT) may be a useful diagnostic modality to identify changes in small intestinal surface temperature related to foreign body obstruction and to identify changes in small intestinal surface temperature related to intestinal bacterial overgrowth (14). Although subjective parameters such as serosal color and mesenteric artery pulsation remain standard, they may not accurately reflect the true degree of mucosal perfusion or microvascular patency. The integration of IRT allowed for objective, real-time quantification of the “rate of change” in surface temperature (21, 22). At T0, the mean surface temperature of the clinically viable segment was 35.1 °C compared with 33.2 °C in the affected segment, corresponding to a difference of 1.9 °C. This difference decreased to 0.4 °C at T1 and 0.1 °C at T2. The lower surface temperature observed in the affected segment at T0 may have been consistent with impaired local perfusion and/or ischemic compromise. However, the progressive reduction in Δ*T* should be interpreted cautiously. In particular, the 0.1 °C difference observed at T2 should not be considered evidence of confirmed or equivalent microvascular perfusion because the measurement accuracy reported for the camera was ±2 °C. An important distinction should also be made between the thermal sensitivity and the measurement accuracy of the IRT device. The reported thermal sensitivity of 0.08 °C at 30 °C describes the ability of the camera to detect small temperature differences under the specified conditions, whereas the manufacturer's reported accuracy of ±2 °C refers to the expected deviation of the measured temperature from the actual temperature. Therefore, the thermal sensitivity should not be interpreted as an indication that absolute temperature measurements were accurate to ±0.08 °C. This distinction is particularly relevant when interpreting the small temperature differences observed at T1 and T2. The present findings should also be interpreted in the context of the absence of validated thermographic reference values or diagnostic thresholds for intestinal viability in horses. The use of a clinically viable intestinal segment from the same animal as a comparative reference may reduce some inter-animal variability; however, this approach does not establish a diagnostic threshold for tissue viability. Accordingly, the present case should be considered an illustration of the potential usefulness of comparative thermal assessment rather than validation of a specific Δ*T* value as a marker of intestinal viability. The thermographic pattern observed in this case may also have been influenced by intraoperative factors. Intestinal manipulation, exposure of the bowel to the operating-room environment, changes in tissue position, ambient temperature, relative humidity, duration of exteriorization, and evaporative cooling can all affect surface temperature independently of tissue perfusion. In the present case, approximately 10 s elapsed between removal of the surgeon's hands from the intestinal surface and acquisition of the thermographic image. This interval was used to minimize the immediate thermal influence of direct manipulation, although it cannot completely exclude the effects of handling or environmental exposure. These factors should therefore be considered when interpreting small changes in surface temperature during intraoperative IRT assessment (23). Furthermore, our observations correlate with the findings of Malafaia et al. (24), in which temperature differentials between uncompromised and compromised intestinal loops in rats with severe diffuse lesions ranged from −1.0 °C to −2.0 °C. In agreement with Moss et al. (19, 20) conclusion that infrared imaging is a reliable tool for identifying necrotic intestinal areas, this case demonstrates the clinical utility of this diagnostic approach in the equine species, particularly for determining the necessity of resection (25). Nevertheless, differences between experimental models and clinical equine surgery, together with differences in camera systems, acquisition protocols, environmental conditions, and definitions of tissue viability, limit direct comparison of numerical temperature thresholds across studies. Further validation in equine patients is therefore required. The interpretation of the initial cold thermal pattern as definitive evidence of microvascular thrombosis is not supported by the present data. Although reduced surface temperature may be compatible with impaired local perfusion or ischemic injury, IRT alone cannot establish the presence of microvascular thrombosis or other specific vascular lesions. Histopathological examination of the resected intestinal margins was not performed in this case, and therefore the thermographic findings could not be correlated with histological evidence of ischemia, necrosis, or vascular injury. This represents an important limitation when considering the diagnostic value of IRT for intestinal viability assessment. The decision to perform intestinal resection and select the anastomotic margins was therefore based on the overall intraoperative evaluation, including conventional macroscopic and clinical criteria, rather than on IRT findings alone. IRT should consequently be considered an adjunctive technique that may provide additional information during surgical assessment rather than a stand-alone method for determining intestinal viability. In this case, the agreement between the conventional assessment and the subsequent thermographic findings was clinically useful, but it does not establish that IRT independently improved the selection of the resection margins or the outcome of the anastomosis. Regarding the underlying etiology of the present case, horses may ingest foreign materials while consuming hay, straw, or forage from different environments. The interval between ingestion and severe symptoms depends largely on the size of the object and the degree of intestinal obstruction; consequently, establishing a direct causal relationship between foreign body ingestion and the subsequent development of colic is often difficult (3, 26–29). Reports of foreign bodies causing perforation or obstructive disease of the small intestine are comparatively rare. Metallic foreign bodies are the most frequently described in the literature (30–34), although a broad range of other materials has also been reported, including rubber, wood, plant material, and porcupine quills (1, 2, 26, 35, 36). The presence clinical relevance of these foreign bodies is often underestimated because diagnosis is often challenging and clinical signs are nonspecific (3, 29). In addition, management conditions, selective feeding behavior, and the difficulty in detecting non-radiopaque materials further complicate diagnosis. The clinical and laboratory findings observed in the present case are consistent with those described by Saulez et al. (4), in which foreign body perforation induced a marked systemic inflammatory response. Interestingly, while the clinical presentation of foreign body ingestion is often inconsistent, our patient exhibited a marked leukocytosis (16,600/mm^3^) and hyperproteinemia (8 g/dL), mirroring the profile of “horse 4” in the referenced study. Similarly, the marked increase in blood lactate was indicative of severe systemic compromise and tissue hypoperfusion but cannot, by itself, establish a specific inflammatory or septic etiology (37). Regarding diagnostic difficulties, while foreign body obstructions in certain segments such as the small colon can sometimes be detected, as reported by Gay et al. (38) who write of a 50% detection rate by rectal palpation, identifying objects located more orally in the jejunum is more difficult, as they are typically not reachable by rectal examination. This anatomical limitation and clinical uncertainty, combined with the progressive severity of abdominal pain, underscore the profound diagnostic difficulties encountered in the present case. Furthermore, Saulez et al. (4) highlight the inherent limitations of abdominal ultrasound in identifying even metallic foreign bodies; such a task becomes significantly more arduous when dealing with wooden objects, as identified in our patient, due to their different echogenic properties and the potential for masking by intestinal gas. A differential diagnosis could also include parasitic impactions. However, in this case, suspicion was low due to the horse's regular deworming and stable management rather than intensive pasturing (39, 40). Postoperative complications, specifically postoperative ileus (POI) and the formation of intra-abdominal adhesions, are well-documented risk factors for small intestinal obstruction in the equine patient (7, 41–43). In the present case, however, a postoperative etiology was definitively excluded based on the subject's medical history. The mare had no prior history of abdominal surgery or exploratory laparotomy, thereby eliminating surgical trauma or iatrogenic fibrin deposition as potential predisposing factors for the current colic episode (41). It must be acknowledged, however, that the absence of specific markers of systemic inflammation (such as serum amyloid A or fibrinogen) and an analysis of peritoneal fluid represents a limitation in our clinical case, preventing a definitive differentiation between non sepsis-related systemic inflammation and local septic involvement, since we relied exclusively on systemic parameters such as leukocytosis and hyperlactatemia. In the case under examination, facial expressions yielded a high score at the time of admission, likely attributable to the animal's pain and discomfort. This score decreased significantly during subsequent monitoring, suggesting the successful resolution of the intestinal obstruction and adequate pain control achieved through the analgesic protocol (44–46). Despite the different limitations, this case highlights the potential role of IRT as a non-invasive and complementary imaging modality during equine abdominal surgery. Its main potential value may lie in providing real-time comparative information on surface thermal patterns that can be interpreted together with established clinical and surgical indicators of intestinal viability. The present findings should therefore be regarded as preliminary and hypothesis-generating rather than as evidence of a validated thermographic method for determining intestinal perfusion or viability. Further studies involving larger case series and standardized protocols are warranted to better define the clinical applicability, diagnostic accuracy, and prognostic value of infrared thermography in equine abdominal surgery.

## 4 Conclusion

Foreign body-associated intestinal perforation should be considered among the differential diagnoses in horses presenting with severe small intestinal colic that fails to respond to appropriate medical therapy, although definitive preoperative diagnosis remains challenging. This case demonstrates that prompt surgical intervention can successfully resolve jejunal obstruction and perforation caused by a wooden foreign body. In addition, intraoperative IRT provided useful additional information on the surface temperature of the examined stumps, supporting surgical decision-making during intestinal resection and anastomosis. By allowing real-time assessment of the temperature difference (delta) between viable and compromised intestinal segments, IRT may be a promising complementary tool to improve surgical precision and potentially reduce the risk of postoperative complications associated with inadequate assessment of intestinal viability.

## Data Availability

The original contributions presented in the study are included in the article/supplementary material, further inquiries can be directed to the corresponding authors.
